# Viroporin potential of the lentivirus lytic peptide (LLP) domains of the HIV-1 gp41 protein

**DOI:** 10.1186/1743-422X-4-123

**Published:** 2007-11-20

**Authors:** Joshua M Costin, Joshua M Rausch, Robert F Garry, William C Wimley

**Affiliations:** 1 Biotechnology Research Group, Department of Biology, Florida Gulf Coast University, 10501 FGCU Blvd. S., Fort Myers, FL, 33965, USA; 2 Department of Neurobiology and Physiology, Northwestern University, 2205 Tech Dr. Hogan 2-160, Evanston, IL 60208, USA; 3 Department of Microbiology and Immunology, Tulane University Health Sciences Center, 1430 Tulane Avenue, New Orleans, LA 70112, USA; 4 Department of Biochemistry, Tulane University, Tulane University Health Sciences Center, 1430 Tulane Avenue, New Orleans, LA 70112, USA

## Abstract

**Background:**

Mechanisms by which HIV-1 mediates reductions in CD4+ cell levels in infected persons are being intensely investigated, and have broad implications for AIDS drug and vaccine development. Virally induced changes in membrane ionic permeability induced by lytic viruses of many families contribute to cytopathogenesis. HIV-1 induces disturbances in plasma membrane ion transport. The carboxyl terminus of TM (gp41) contains potential amphipathic α-helical motifs identified through their structural similarities to naturally occurring cytolytic peptides. These sequences have been dubbed lentiviral lytic peptides (LLP) -1, -2, and -3.

**Results:**

Peptides corresponding to the LLP domains (from a clade B virus) partition into lipid membranes, fold into α-helices and disrupt model membrane permeability. A peptide corresponding to the LLP-1 domain of a clade D HIV-1 virus, LLP-1D displayed similar activity to the LLP-1 domain of the clade B virus in all assays, despite a lack of amino acid sequence identity.

**Conclusion:**

These results suggest that the C-terminal domains of HIV-1 Env proteins may form an ion channel, or viroporin. Increased understanding of the function of LLP domains and their role in the viral replication cycle could allow for the development of novel HIV drugs.

## Background

The two noncovalently associated envelope glycoproteins, surface (SU) and transmembrane (TM), of HIV-1 are responsible for attachment and entry into target cells. SU, or gp120, is entirely extracellular and contains the motifs responsible for cell receptor recognition and attachment, among others. TM, or gp41, contains the transmembrane anchor domain responsible for anchoring the envelope protein of the virion into membranes, as well as the fusion domain which is responsible for entry into cells through fusion of the viral and cellular lipid membranes. TM contains several additional functional domains, including the lentivirus lytic peptide (LLP) domains. These domains were identified on the basis of their structural motifs and similarities to several natural cytolytic peptides [1]. One such cytolytic peptide, magainin-2, was discovered after a biomolecular search of the mucosal surfaces of the *Xenopus laevis *frog. It was later shown to possess broad spectrum anti-bacterial activity that is due to microbial membrane permeabilization [1-4]. Magainin-2 is also hemolytic, but at concentrations 1–3 orders of magnitude higher than is needed for bactericidal activity [5]. Analysis using the patch clamp technique identified magainin-2 as a voltage-dependent ion channel [6].

Biochemical analyses yielded insights into the mechanism of action of magainin-2. This peptide is cationic, amphipathic, and adopts an α-helical secondary structure in the presence of lipid [5,7]. Molecular modeling studies supported by experimental evidence suggested that the activity of magainin-2 is tied to its ability to form a multimeric structure after insertion into lipid membranes [8,9]. Similar structure-function relationships have been discovered for other natural lytic peptides, such as the cecropins of the North American silk moth, *Hyalophora cecropia*, and melittin from the venom of the honey bee, *Apis mellifera *[9,10].

Experimental evidence suggests that similarities between previously identified natural cytolytic peptides and the lentivirus lytic peptides are more than speculative. Circular dichroism and FTIR studies suggest that peptides corresponding to all three LLP domains adopt amphipathic α-helical secondary structure in the presence of lipid environments of differing composition [11-14]. LLP-1 and -2 cause the release of carboxyfluorescien entrapped in phosphatidyl choline (PC) vesicles [11]. 15-mer peptides overlapping the LLP-1 and -2 domains of a concensus B clade virus were able to rupture large unilamellar vesicles (LUV's), as well as induce phospholipid mixing and fusion of LUV's [15]. Functionally, LLP-1 and -2 can lyse bacteria, fungus, red blood cells, and various cultured eukaryotic cells [1,11,16-21]. LLP-1 has been shown to increase the conductance of both planar lipid bilayers and *Xenopus *oocytes, presumably caused by the formation of transmembrane pores which increase the membrane permeability of electrogenically active ions [22,23]. Based on available evidence, it has been postulated that LLP-1, and possibly LLP-2 peptides, oligomerize to form a "barrel-stave"-like pore, which are conducting pores (barrels) in membranes formed by the self-assembly of a variable number of alpha-helical rods (staves).

Formation of ion channels could subsequently allow ions to be redistributed across the membrane. Increases in intracellular ion concentrations, followed by water for osmotic balance have been postulated to cause cell volume dysregulation observed with infected CD4^+ ^T lymphoblastoid cells *in vitro *[24-27]. Syncytial cells as well as singly infected cells show increases in cell volume. The latter can undergo a process termed "balloon degeneration" in which an irreversible expansion of cell volume occurs beyond the limits of cell membrane integrity, resulting in osmolysis. The ability of UV-inactivated HIV to cause single cell balloon degeneration in the absence of replication argues for the involvement of a virion component, possibly the LLP domains [28].

The same case has not been made for LLP-3 as has been made for LLP-1 and 2 however. Synthetic LLP-3 peptides partition into small unilamellar vesicles (SUV's) containing phosphatidyl choline (PC), as evidenced by an increase in quantum yield and a blue shift in the emission maximum of the intrinsic tryptophan fluorescence, but do not appear to span the membrane [14]. Concomitantly, negative staining electron microscopy of LLP-3 exposed PC vesicles shows a disrupted membrane without the formation of a pore. Sequence analysis and modeling of LLP-3 predicts a leucine zipper-like motif in place of the repeated charged residues found on the hydrophilic surface of LLP-1 and -2. This discovery has led to the theory that the LLP-3 domain of TM may play a role in oligomerization of the TM tail containing the LLP domains based on the roles of previously identified leucine zipper motifs, including one in the ectodomain of TM [14,29].

The experiments below represent the first direct comparison of all three LLP domains. We demonstrate that synthetic peptides corresponding to the three LLP domains are capable of partitioning into POPC:POPG membranes, and in doing so adopt a more ordered amphipathic α-helical secondary structure. Furthermore, as a consequence of partitioning into POPC:POPG membranes in an α-helical conformation, peptides corresponding to all three LLP domains are able to disrupt lipid membranes in the absence of any other proteins, cellular or viral, though the manner by which these three regions interact with membranes may vary.

## Results

### LLP domains form amphipathic α-helices

**Figure 1:**
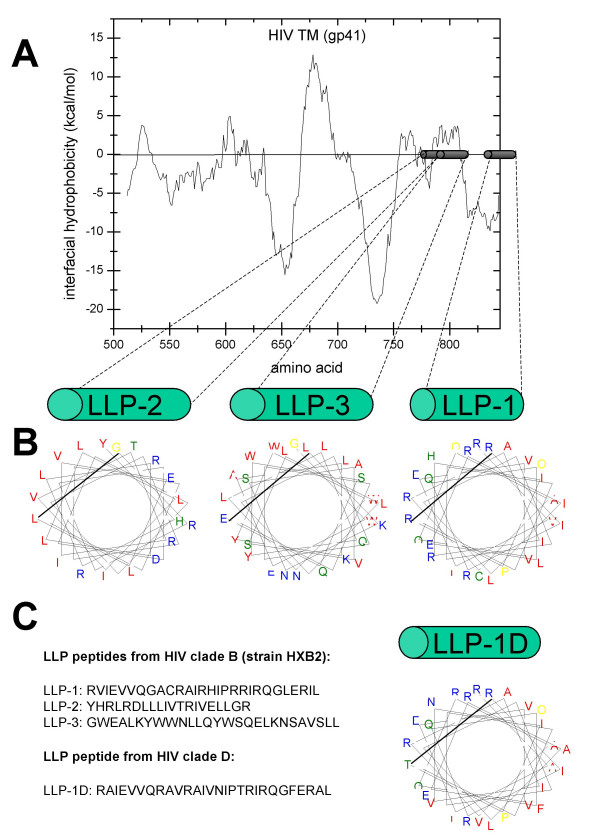
**Sequence and predicted secondary structures of the LLP domains**. (A) Wimley-White hydrophobicity plot of TM predicted amino acid sequence of HIV_HXB2_. Highlighted cylinders show the locations of the LLP domains. A hydrophibicity score could not be calculated for the entire LLP-1 domain due to its location at the extreme c-terminus of TM. (B) Helical wheel diagrams showing the amphipathic nature of each LLP domain. The coloring scheme is from Benner et al. and graphs were generated using a java applet [72]. (C) Primary amino acid sequence of the synthesized peptides used in subsequent experiments which correspond to the LLP-1, -2, and -3 domains of the TM protein.

Three domains have previously been identified in the C-terminus of TM from HIV-1 strain HXB2 (clade B) with homology to natural lytic peptides, such as magainin-2, and to the S4 domain of K^+ ^and Na^+ ^channels [12,14,30]. These domains, identified as LLP-1, LLP-2, and LLP-3 for the order of their discovery, were examined on the Wimley-White (W-W) interfacial hydrophobicity scale for their propensity to partition in lipid membranes (Figure 1A). The W-W hydrophobicity scale is the first experimentally determined hydrophobicity scale based on the transfer of free energies for each amino acid [31]. This scale takes into account contributions from the peptide bonds and side chains when partitioning into membranes. A W-W score greater than zero indicates a propensity to partition into lipid membranes. LLP-3 scored the highest average interfacial hydrophobicity, +3.26 kcal/mol, and is predicted to partition into membranes. LLP-2 possessed an average hydrophobicity score of 0 kcal/mol and based on this score alone LLP-2 would not be expected to partition into membranes. Likewise, LLP-1 would not be expected to partition into membranes with an average interfacial hydrophobicity score of -8.42 kcal/mol.

The mean hydrophobicity scores for LLP-1, -2, and -3 are based only on primary amino acid sequence, and do not take into account contributions from higher order structure. It has recently been shown that membrane binding of helical peptides is driven much more by amphiphilicity than by overall hydrophobicity [32]. Figure 1B contains helical wheel diagrams of each LLP domain. When plotted as α-helices, it is apparent that all three domains are amphipathic, generally with hydrophilic residues (colored blue) clustered on one face of the α-helix and hydrophobic residues (colored red) clustered on the opposite face. LLP-3 differs from LLP-1 and -2 in that it lacks the positively charged residues on its hydrophilic face. This secondary structure is conserved across HIV-1 clades, though primary amino acid sequence identity is not, suggesting that this structure is important for the virus [1]. For comparison, the primary amino acid sequence of an LLP-1 domain from a clade D HIV-1 virus, named LLP-1D for the purposes of the present studies, (Fig. 1C) and helical wheel diagram (Fig. 1B) are shown.

**Figure 2:**
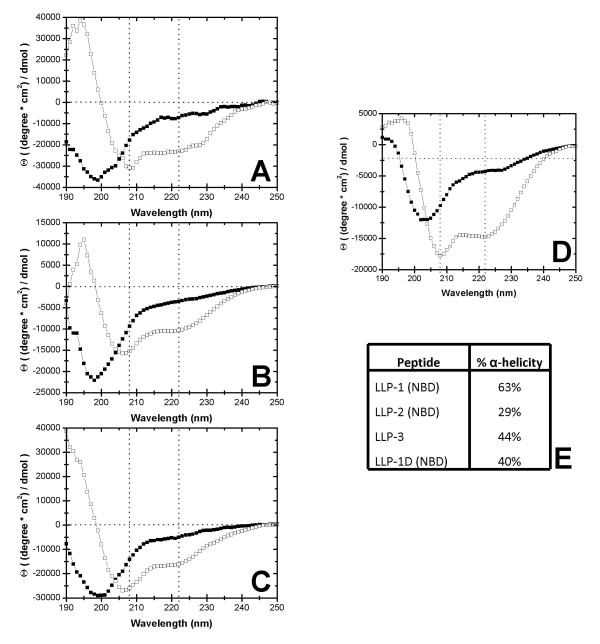
**LLP peptides form α-helices in the presence of lipid**. Circular dichroism spectroscopy of LLP peptides in PO_4 _buffer (open squares) and in the presence of 90%POPC:10%POPG (filled squares). Spectroscopic analysis revealed that each peptide possessed the characteristic minima at 208 nm and 222 nm indicating α-helical character. (a) LLP-1 labeled with NBD; (b) LLP-2 labeled with NBD; (c) LLP-3; (d) LLP-1D labeled with NBD. (e) The percent α-helicity was calculated from the CD spectroscopy curves in figure a-d for each peptide using the following formula: Θ_222_/(1–2.5/n)(40,000), where n = the number of residues present in the peptide.

Peptides were synthesized from the primary amino acid sequences given in Figure 1C. Fluorescent NBD (4-chloro-7-nitrobenz-2-oxa-1, 3-diazol) labels were attached to the N terminus of peptides lacking tryptophan residues (LLP-1, LLP-2, and LLP-1D) for lipid membrane partitioning and circular dichroism experiments, as well as for quantification purposes. Experimental evidence exists suggesting that peptides corresponding to these domains adopt α-helical secondary structure in the context of some lipid environments [11-14]. Figure 2 shows the circular dichroism spectra of peptides corresponding to each of the three LLP domains in the presence (unfilled squares) and absence (filled squares) of lipid vesicles composed of 10% POPG: 90% POPC. LLP-1 peptide in the presence of KPO_4 _buffer alone gave the characteristic spectrum of a randomly ordered peptide. After the addition of 10% POPG: 90% POPC LUVs, a shift towards a more ordered structure was observed, with minima at 208 nm and 222 nm (vertical dashed lines) corresponding to characteristic α-helical spectra. Similar results were observed with peptides corresponding to the LLP-2 (Fig. 2B), LLP-3 (Fig. 2C), and LLP-1D (Fig. 2D) domains, where dramatically enhanced α-helical secondary structure was observed in the presence of a membranes. The percent α-helicity was calculated from the Θ_222 _value observed in the above spectra and the results are presented in figure 2E.

### LLP-1, -2, and -3 partition into lipid bilayers

**Figure 3:**
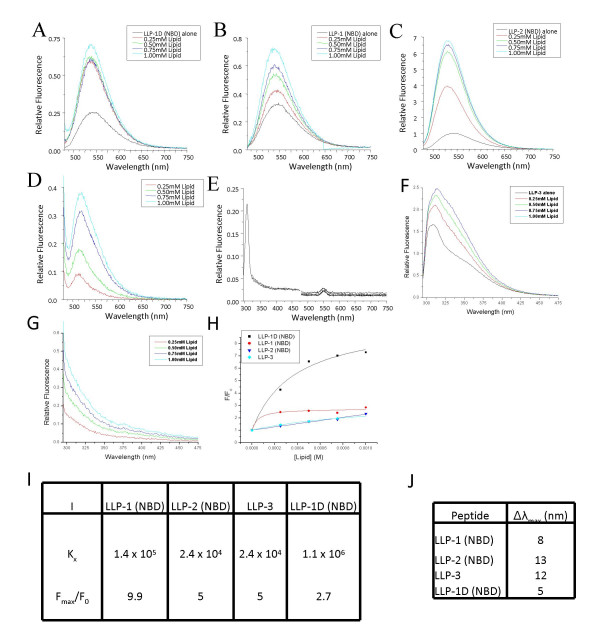
**LLP peptides partition into lipid bilayers**. Fluorescence enhancement of tryptophan or NBD with partitioning of LLP peptides into lipid bilayers (a) LLP-1 (NBD), (b) LLP-2 (NBD), (c) LLP-1D (NBD), (d) 10%POPG:90%POPC (NBD) lipid alone controls (e) PO_4 _blanks (f) LLP-3, (g) 10%POPG:90%POPC (tryptophan) lipid alone controls. Partitioning of LLP peptides partitioning into lipid bilayers are presented as fluorescence enhancements in (h) and the results of curve fitting are shown in (i). In (j), the largest blue shift of the emission maxima for each peptide indicating transitions from aqueous to lipid environments are shown. A, B, C, and F represent lipid blank and PO_4 _blank subtracted spectra. H, I, and J are all calculated from these normalized spectra.

Each LLP peptide was assayed for its ability to interact with the lipid membranes of the same lipid composition as those used for the CD spectroscopy. In a low-polarity environment, such as the lipid membrane interface, the fluorescence of tryptophan and NBD increases in quantum yield and shifts the emission maximum to shorter wavelengths. Thus by observing the change in tryptophan or NBD fluorescence (F) as a function of increasing lipid concentration, the degree to which a peptide partitions into a lipid membrane can be determined. The fluorescence spectra for each LLP peptide and accompanying controls are presented in Figure 3A–G. An enhancement of fluorescence is observed with all four peptides tested after the addition of increasing lipid titrations indicating membrane partitioning. Fluorescence intensities are presented as a function of increasing lipid concentration for all peptides in Figure 3H. The intensity plateau for LLP-1 and LLP-1D peptides upon lipid titration indicates that these peptides are nearly fully bound at the highest lipid concentrations, while the monotonic increase and low overall enhancement of LLP-2 and LLP-3 indicates that they are only partially bound at these lipid concentrations. The difference in fluorescence enhancement between LLP-1 and LLP-1D does not indicate a difference in partitioning but rather a difference in the environment of the probe after partitioning.

From the fluorescence intensities in Figure 3H, partition coefficients for each peptide can be estimated (Materials and Methods). Calculated partition coefficients and fluorescence enhancements are shown in Table 3I. A blue shift of the emission maxima (Figure 3J) further corroborates that the peptides are entering the hydrophobic environment of the lipid membrane from the aqueous solution. The manner in which each peptide interacts with the membrane, either lying on the surface or spanning the membrane as an aggregate to form a pore can not be directly determined from this data.

### LLP-1, -2, and -3 disrupt large unilamellar vesicles

**Figure 4:**
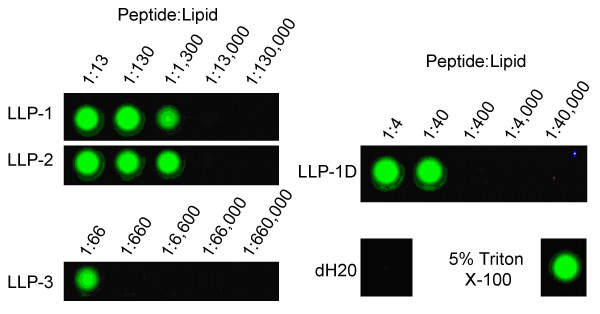
**LLP peptides disrupt lipid membranes**. Tb3+/DPA assay for peptide induced membrane permeation. Disruption of 90%POPC:10%POPG large unilamellar vesicles (LUV's) containing entrapped terbium and external DPA by LLP peptides is indicated by green Tb/DPA fluorescence under UV illumination.

The Tb^3+^/DPA assay, a high throughput leakage assay developed by Rausch and Wimley, 2001, was employed in order to determine each LLP peptide's ability to disturb lipid membrane integrity. This technique relies upon the greatly increased fluorescence emission that occurs when the lanthanide metal terbium interacts with the aromatic chelator dipicholinic acid (DPA). To this end, Tb^3+ ^was entrapped in Large Unilamellar Vesicles composed of 10% POPG: 90% POPC diluted in a KPO_4 _buffer containing DPA. Only upon membrane disruption was terbium able to come into contact with DPA in the buffer generating a fluorescent complex. Various ratios of peptide:lipid were incubated together in a microwell plate and the resulting fluorescence emissions were monitored under UV irradiation. LLP -1, -2, and -1D peptides used in this study were not NBD labeled, but were N-terminally labeled with a tryptophan residue for quantification. The results are presented in Figure 4. LLP-1 and -2 disrupted LUV's at very low peptide:lipid ratios of approximately 1:1,300. Roughly 20 and 32 times as much peptide was required to induce leakage from vesicles with LLP-3 and LLP-1D respectively. Complete dissolution of membranes with Triton X-100 shows 100% leakage of vesicles versus virtually no leakage with distilled water. The known pore forming antibiotic alamethacin was used as an additional positive control and produced similar results in the assay as Triton X-100 (data not shown).

## Discussion

In good agreement with the literature, the present set of experiments confirms that the LLP domains present in the TM portion of the Env protein of HIV-1 form amphipathic α-helical structures in the presence of a 10% POPG: 90% POPC lipid environment. Each of these peptides were able to bind to and disrupt membranes of this composition, despite a lack of amino acid sequence identity. The presence of an NBD tag did not appear to affect the biochemical characteristics of the peptides to which it was attached. These experiments represent the first direct comparison of all three LLP domains' interactions from the same HIV-1 virus – HXB2 from clade B – with identical lipid membranes. Additionally, it is the first example of a direct comparison of structure and function of an entire LLP-1 domain from the laboratory adapted HXB2 strain of HIV-1 (i.e., LLP-1) with a natural sequence variant from a clade D HIV-1 virus (i.e., LLP-1D) under identical conditions.

Based on similarities to other amphipathic α-helices, such as magainin-2, it has been hypothesized that the LLP domains could insert into bilayers and form a pore with their hydrophobic faces oriented towards the lipid bilayer and the hydrophilic faces oriented towards the lumen of the newly formed pore [8,9]. The results presented here are consistent with this hypothesis. LLP-1, -2, and -3 domains partition into membranes as an α-helix and disrupt the membrane. The methodologies used here are not able to distinguish between membrane insertion and interactions at the membrane-water interface in which the peptides lie on the cell surface to cause a generalized disruption of the membrane. However, our observation of nearly complete leakage from vesicles at P:L ratios exceeding 1:1000 for LLP-1 and -2 supports the idea of a membrane-spanning pore. Such high activity has not been observed for surface-active membrane-spanning pore. Such high activity has not been observed for surface-active pore-forming peptides which generally cause 100% leakage at P:L around 1:50 [33], whereas barrel-stave peptide pores with the observed level of potency have been described in the literature [34].

Previous studies have sought to define the size of the pore created by LLP-1 peptides alone. Miller et al., 1993 measured the amounts of ^45^Ca, ^14^C-sucrose, and ^14^C-inulin that were able to enter LLP-1 treated CEM cell cultures. ^45^Ca (MW 45 Da) and ^14^C-sucrose (MW 342.3 Da), but not ^14^C-inulin (MW ~5000 Da) were able to pass through LLP-1 treated membranes, but not untreated CEM cells [21]. In good agreement, membrane perturbation studies utilizing whole virus show that hygromycin b (MW 527 Da) was able to enter cells after infection with HIV-1, while the similar sized G418 (MW 496 Da) was not able to enter [35]. This suggests that the pore created by the LLP domains has a cutoff around MW = 500 Da.

LLP-3 forms an amphipathic α-helix in the presence of lipids and binds to lipids and disrupts lipid vesicles, but lacks the overall positive charge of the LLP-1 and -2 domains. Kliger et al., 1997 originally identified a leucine zipper-like sequence on its hydrophilic face [14]. The authors proposed that this type of domain is likely useful in oligomerization of the cytoplasmic tails. This is analogous to an amphipathic α-helical/leucine zipper-like sequence in the TM ectodomain already proposed to play a role in Env oligomerization [36,37]. Whether this LLP-3 mediated oligomerization takes place through spanning the membrane, on the inner surface of the membrane, or not at all is unknown and will require characterization of the domain in the context of the protein. LLP-3 is additionally suspected to contain at least one region that interacts with the matrix protein of the virus, both in the virion and in the infected cell [38].

It is possible that the membrane lipid composition could affect results in the types of studies presented here. There is recent precedent for this in the virus literature, including the HIV-1 literature [39]. The presence of sphingomyelin in LUV's exposed to 15-mer peptides overlapping the LLP domains increased membrane disruption as well as lipid mixing and fusion activities [15]. An attempt was made to perform the above experiments in a different vesicle composition (18%PE : 65%PC : 10%PI : 2%PS : 5%SM and cholesterol/PL (mol/mol) of 0.5). This composition incorporates SM and reflects the basic lipid composition of *Xenopus laevis *oocytes and would have allowed for a more direct comparison to physiological experiments to be performed with the same peptides in that system [40,41]. Unfortunately, LUV's of this composition were inherently unstable and unusable (data not shown). Therefore, the simpler vesicles composed of 10% POPG: 90% POPC were utilized as a reasonable mimic of the thickness, fluidity and electrostatic surface potential of a biological membrane. It has been previously shown that the positive charge of the LLP peptides are important for its ability to interact with negatively charged lipid membranes [19,21]. Thus the use of the negatively charged POPG was appropriate for these studies defining the structure of these domains while binding to an anionic membrane surface.

**Figure 5:**
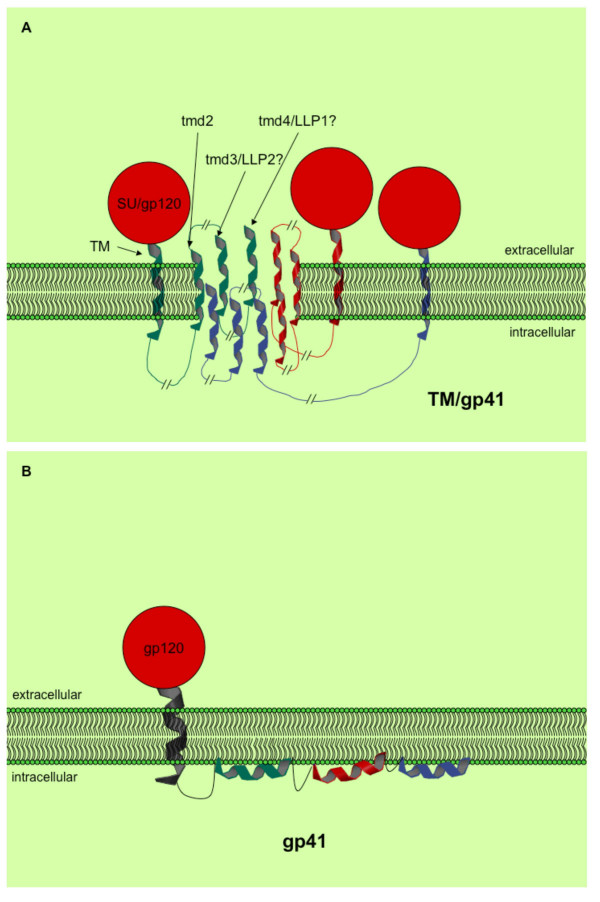
**Proposed models of the C-terminus of TM**. Proposed models of LLP domains in the context of TM and in a lipid membrane. a) A nine pass transmembrane configuration and b) Association of the LLP domains with the inner leaflet of the lipid membrane allowing for interaction with calmodulin. It is possible that the LLP domains flip-flop between this configuration and a transmembrane configuration.

Integrating the current biochemical and physiological data gathered using the lentiviral lytic peptides, a hypothetical model of their action in the membrane is proposed in Figure 5. The LLP regions of TM are α-helical in a lipid environment, partition into lipid bilayers, and disrupt lipid membranes. Since Env is known to associate in trimers on the cell surface and in virions [42,43], it is easy to speculate that the LLP regions of the Env trimers could associate with each other, forming a pore or channel in the area between them.

Figure 5A depicts one possible configuration of a pore formed by the cytoplasmic tail and LLP regions of gp41 (TM). Further support for this transmembrane configuration of the cytoplasmic tail of TM has come from the detection and characterization of neutralizing antibodies to several regions of the Kennedy peptide, a very hydrophilic region spanning approximately residues 731–752 of the cytoplasmic tail of TM (between TMD2 and TMD3 in Figure 5A) [44-48]. Cleveland et al, 2000 suggest that the major TM domain of gp41 actually span the membrane twice (labeled as TM and TMD2 in Figure 5A). This could allow the TMD3 and TMD4 to be LLP-2 and LLP-1 respectively, placing LLP-3 on the inner leaflet of the plasma membrane to interact with the matrix protein. However, direct evidence for this model is currently lacking, leaving open the possibility of an as of yet unidentified membrane spanning region that would constitute TMD2.

The presence of the hydrophilic region, or Kennedy peptide, outside the membrane suggests that there would need to be at least one additional membrane spanning domain to bring the rest of the cytoplasmic tail back into the interior of the cell. This could make the environment more favorable to additional membrane spanning regions, such as LLP-1 or even LLP-3.

Based on its average W-W hydropathy score, LLP-3 may lie on the surface of the inner leaflet of the plasma membrane. LLP-3 domains in this case may interact with each other through the leucine zipper-like motifs formed from the peptide's α-helical secondary structure and/or the LLP-3 domain could then be free to interact with the matrix protein of HIV [38,49]. This could have the effect of stabilizing the Env trimers and/or the resulting transmembrane pore that could then be formed by the LLP-1 and -2 domains. The location of the LLP-3 domain on the inner leaflet of the cell membrane could also serve to nonspecifically destabilize the lipid bilayer to increase viroporin function as has been observed with Simliki forest virus domains [50].

Viral ion channels, or viroporins, are present in many lytic animal viruses. Increased membrane permeability caused by viroporins, glycoproteins, and proteases is a typical feature of animal virus infections [51]. Viroporins are virally encoded, small (generally ≤ 120 amino acid residues) membrane proteins that form selective channels in lipid membranes. Features common to viroporins include: promoting the release of virus, altering cellular vesicular and glycoprotein trafficking, and increasing membrane permeability. Amphipathic α-helical domains of viroporins generally oligomerize to form the channel by inserting into lipid membranes with the hydrophobic residues oriented towards the lipid bilayer and the hydrophilic residues facing in towards the lumen of the channel. Though viroporins are not essential for virus replication, they may be necessary for full pathogenesis *in vivo *as they are known to enhance virion production and release [52-54]. Mounting evidence, including data presented here, suggests that the intracellular tail of gp41 constitutes a viroporin and deserves further investigation as such to determine its exact role in the viral replication cycle.

That HIV may code a viroporin in its major surface glycoprotein would ensure that the membrane perturbation, ion fluxes, volume changes, and resulting "loosening" of the plasma membrane and cytoskeleton always occur when and where it is needed for budding, syncytial formation, and/or single cell balloon degeneration. Concentrating HIV glycoproteins in lipid rafts could allow for localized unstable membrane regions at the exact points where it is needed by HIV. While it seems possible that Vpu could also act at these stages to accomplish the same goals, given that it also causes membrane leakage, it is more difficult to envision how it could accomplish the task as Vpu has been shown to be excluded from the plasma membrane and HIV virions [53,55].

Prior observations that LLP-1 can bind to intracellular signalling molecules, such as calmodulin to ultimately induce apoptosis and/or necrosis [16,18,19,56] suggest that the LLP domains may be configured in certain situations to be associated exclusively with the inner leaflet of the lipid membrane where they are able to interact with these intracellular molecules (see Figure 5B). Flip-flopping between lipid bilayers of amphipathic pore forming peptides has been documented with melittin [57,58]. Based on reported similarities between melittin and LLP peptides, it is reasonable to hypothesize that the LLP domains may be flip-flopping between a transmembrane state and parallel association with the inner leaflet of the lipid bilayer. On the other hand, the LLP domains may possess different activities in the different cell types that it infects, or there may be some as of yet undefined temporal control that allows these two alternate functions to take place at appropriate times during infection.

## Conclusion

Based on these models and on the number of Env proteins known to associate with each virus, an educated guess of the maximum number of pores present in each virion can be deduced. There are approximately 72 Env proteins per virion [59,60]. If 3 Env proteins indeed form a viral pore, based on the proposed trimer arrangement of Env proteins [42,43,61], this would result in 24 viroporins per virion.

Since the LLP domains are also present in the context of the virion, it is possible that they would have an effect at this stage of the HIV replication cycle. There is at least one report of an increase in natural endogenous reverse transcription (NERT) cause by the LLP domains increasing the virion envelope permeability to dNTP's [62].

In addition to the LLP's involvement as a backup system for cell volume regulation and cytoskeletal disruption, they may produce secondary effects, such as AIDS-related dementia complex and bystander cell death. LLP domains could be cleaved by cellular proteases from the C-termini of TM proteins and act as exogenous peptides *in vivo*. In this way they could produce the effects generated by LLP in cell culture thought to cause AIDS-related dementia [63,64]. An analogous role could be played in the death of bystander cells – a population of cells that die in HIV-infected individuals, but are not productively infected [65,66].

In 2004 alone it was estimated that there were approximately 39.4 million people living with HIV/AIDS, with around 3.1 million AIDS related deaths, and 13,500 new infections each day [67]. Even with the advent of Highly Active Anti-Retroviral Therapy (HAART), which combines the use of protease inhibitors and reverse transcriptase inhibitors, and use of the newer fusion inhibitors such as T20, HIV continues to be a serious threat to world health [68,69]. A lack of resources for most infected persons to purchase the drugs, the intensive treatment regimen, the toxicity of drug regiments, and emerging drug resistance all contribute to a lack of general efficacy of the current treatment regimen and highlight the necessity for more basic research with the ultimate goal of development of new treatments. The LLP domains may represent a new target for HIV drugs to inhibit HIV infection. Otherwise the development of eLLP's (engineered LLP's) as a new class of antibacterial drugs could be used to help resolve AIDS-related infections, as well as serve as a new class of antibiotics – virally derived antibiotic peptides.

## Methods

### Peptide Synthesis

Peptides were synthesized by solid-phase synthesis methodology using a semi-automated peptide synthesizer and conventional N-alpha-9-fluorenylmethyloxycarbonyl (Fmoc) chemistry. LLP-3 peptides were synthesized by Genemed Synthesis, Inc. (San Francisco, CA). Peptides were purified by RPHPLC, and their purity confirmed by amino acid analysis and electrospray mass spectrometry. All other peptides were synthesized by Louisiana State University Health Sciences Center core laboratory facility (New Orleans, La). Peptides were purified by HPLC, and their purity confirmed by MALDI mass spectroscopy.

Peptide stock solutions were prepared in dH_2_O, and concentrations were determined spectroscopically (SmartSpec™ 3000, BioRad, Hercules, CA) for those peptides containing one or more tryptophan residues or an NBD label. Concentrations of peptides lacking a tryptophan or NBD label were determined using BCA Protein Assay Reagent Kit (cat. #23227, Pierce Biotechnology, Inc.) according to the manufacturer's protocol. Briefly, colorimetric reagents were mixed for 1 minute, and then peptide samples were added and incubated 30 minutes prior to being read at 562 nm. Concentrations were determined by comparison to a standard curve generated using known BSA concentrations.

### Proteomics Computational Analysis

Predicted amino acid sequence for HIV_HXB2 _was accessed from GenBank (accession # U08446) and analyzed on the Wimley/White hydrophobicity scale using the MPEx (Membrane Protein Explorer) utility available at [70,31]. Resulting hydrophobicity scores were then plotted using Origin 6.0. Average hydrophobicity scores are reported as the hydrophobicity score of the median residue over the selected set of amino acids. Due to its location at the extreme C-terminus of TM, the LLP-1 domain could only be scored over the 16 amino acids out of 30 present in the complete domain. However, a score for the median amino acid of the domain could still be determined and is the score reported as the average hydrophobicity for LLP-1.

Helical wheel diagrams were generated utilizing the coloring scheme from Benner et al. [71]. Helical wheels were generated using a java applet [72]. The data was subsequently graphed in Origin 6.0 to give final helical wheel diagrams.

### LUV Preparation

Large unilamellar vesicles consisting of 90% POPC with 10% POPG (*mol:mol*) (Avanti Polar Lipids, Birmigham, AL) were prepared according to the extrusion method of Nayar, et al. [73,74]. Briefly, lipids were dried from chloroform solution with nitrogen gas stream and high vacuum overnight. Lipid vesicles used in peptide binding assays and CD experiments were resuspended in 10 mM KPO_4 _buffer to bring the lipid concentration to 100 mM. Samples were subjected to repeated freeze and thaw for 15 cycles followed by extrusion through 0.1 μm polycarbonate membranes in a Lipex Biomembranes extruder (Lipex Biomembranes, Vancouver, BC).

To prepare Tb^3+ ^LUVs, lipids were resuspended to 100 mM concentration in 50 mM Tb^3+^, 100 mM sodium citrate, 10 mM TES pH 7.2. Gel Filtration on Sephadex G-200 was used to elute LUVs and remove unencapsulated Tb^3+ ^in a buffer of 10 mM TES and 325 mM NaCl [75]. Final lipid concentrations were determined by phosphate analysis [76,77]. Briefly, lipids were heated in H_2_SO_4 _for 45 minutes, cooled, then heated twice in H_2_O_2 _– first for 30 minutes and then for 90 minutes. Finally, samples were heated in a water bath with AmMo and ascorbic acid for 10–15 minutes and absorbance at 660 nm was measured. Concentrations were determined by comparison to a standard curve generated using known KPO_4 _concentrations.

### Peptide Binding Assay

Partitioning of peptides into lipid bilayers was monitored by the fluorescence enhancement of tryptophan [78] or NBD. For tryptophan, fluorescence was recorded at excitation and emission wavelengths of 280 and 340 nm, respectively, and 8 nm bandwidths using an SML Aminco 8100 spectrofluorometer (Rochester, NY). For NBD label, fluorescence was recorded at excitation and emission wavelengths of 465 and 530 nm, respectively, and 8 nm bandwidths using an SML Aminco 8100 spectrofluorometer (Rochester, NY). Quartz cuvettes were used with excitation and emission path lengths of 4 and 10 mm. Measurements were carried out in a buffer of 10 mM potassium phosphate, pH 7.0. Peptides were added from stock solutions to 250 μl of buffer and mixed by inversion to a final concentration of 2.5 μM. LUVs were titrated into each peptide up to a final concentration of 1 mM. Fluorescence intensities (F) were adjusted for lipid scattering and normalized to peptide in buffer alone (F_o_). Partitioning coefficients were obtained by fitting the formula;

F/F_o _= 1 + (((K_x _× [L])/([W] + (K_x _× [L]))) × ((F_max _/F_o_) - 1)

where K_x _is a mole fraction partition coefficient that represents the amount of peptide in bilayers as a fraction of total peptide present in the system, F_max _is a variable value for the fluorescence enhancement at complete partitioning determined by fitting the equation to the experimental data, [L] is the concentration of lipid and [W] is the concentration of water (55.3 M). The fraction of peptide bound in any experiment can be calculated using the equation

Fraction bound = (K_x _× [L])/([W] + (K_x _× [L]))

In the case of LLP-2 and LLP-3, F_max _can not be determined from the data, so we estimate it to be ~5, based on our experience with NBD-labelled peptides, in order to estimate partition coefficients.

### Tb^3+^/DPA microwell plate assay

For the microwell plate assay, a 200 μl aliquot of vesicle solution containing 500 μM Tb^3+ ^LUV solution in 10 mM TES, 50 uM DPA, 325 mM NaCl, pH 7.2 was pipetted into each well of a plastic 8 × 12 format plate [75]. Peptides were added to each well, thoroughly mixed, and plates were allowed to incubate at room temperature for 2 hours. In addition to peptide-treated wells, dH_2_O and Triton-X-100-treated (Sigma, St. Louis, MO) wells served as negative and positive controls, respectively. After 2 h incubation, Tb^3+^/DPA fluorescence was visualized under a horizontally mounted short-wave (254 nm) UV light source in a darkroom [75]. Plates were photographed and images recorded on a Nikon Coolpix 995 using a 4 sec exposure time with 100 speed, 2.6 aperture and a 540 nm band pass optical filter between sample and lens. For each experimental plate, we directly compared the Tb^3+^/DPA fluorescence of the peptide-treated wells to that of wells containing the same amount of untreated vesicles and to wells containing vesicles that had been lysed with the detergent Triton X-100. Color adjustment and contrasting were normalized to negative controls using Adobe Photoshop.

### Circular Dichroism

CD spectra were recorded using a Jasco J-810 spectrapolarimeter (Jasco Inc., Easton, MD). The sample was contained in a 1 mm path length cell at room temperature. The CD data are expressed as the mean residue ellipticity. All CD runs were made with peptide dissolved in 10 mM potassium phosphate buffer at pH 7.0. Lipids were titrated to 1 mM from a stock in 10 mM phosphate.

### Data Analysis

Diagrams were made using a combination of Microsoft Powerpoint and Adobe Photoshop. Peptide binding and CD spectroscopy data were graphed and analyzed using Origin (Northampton, MA).

## Abbreviations

AmMo : Ammonium Molybdate;

BSA : Bovine serum albumen;

HPLC : high performance liquid chromatography;

DPA : 2,6 Pyridinedicarboxylic acid;

LUV : Large unilamellar vesicle;

NBD : 4-chloro-7-nitrobenz-2-oxa-1, 3-diazol;

POPC : 1-Palmitoyl-2-oleoyl-sn-glycero-3-phosphatidylcholine;

POPG : 1-Palmitoyl-2-oleoyl-sn-glycero-3-phosphatidylglycerol;

RPHPLC : Reversed phase high performance liquid chromatography;

SUV : Small unilamellar vesicle;

Tb : Terbium(III) chloride hexahydrate

## Competing interests

The author(s) declare that they have no competing interests.

## Authors' contributions

JMC performed all experiments with substantial help from JMR. RFG and WCW provided guidance, expertise, equipment, and funding for these experiments. All authors have read and approved this manuscript.
